# Supplementary material for: Establishment and validation of a risk prediction model for adverse drug reactions in patients with coronary heart disease after taking statins: a retrospective study
Source: PeerJ. 2025 Jul 1;13:e19630. doi: 10.7717/peerj.19630 (PMC12226984; doi:10.7717/peerj.19630)
Supplement: Supplemental Information 3 [file peerj-13-19630-s003.docx]

STROBE Statement—checklist of items that should be included in reports of observational studies

|  | Item No. | Recommendation | Page  No. | Relevant text from manuscript |
| --- | --- | --- | --- | --- |
| **Title and abstract** | 1 | (*a*) Indicate the study’s design with a commonly used term in the title or the abstract | 2 | A retrospective cohort study was employed to gather clinical data from a sample size of 351 individuals diagnosed with coronary heart disease who have been diagnosed with coronary heart disease and received statin treatment at the cardiology department of a tertiary hospital located in Anhui Province, China, from February 2021 to January 2022. |
|  |  | (*b*) Provide in the abstract an informative and balanced summary of what was done and what was found | 2 | The clinical data of 351 subjects were partitioned into two groups, namely the development group consisting of 283 cases and the validation group comprising 68 cases, following an 8:2 ratio randomly. In the development group, Logistic regression was employed to identify independent risk factors associated with adverse drug reactions induced by statins in patients diagnosed with coronary heart disease. The R software was employed to construct a nomogram prediction model for the identified independent risk factors, and the clinical applicability, discrimination, and calibration of the model was analyzed. Results: The incidence of ADR in 351 subjects was 24.22%, which was divided into three categories based on the location of ADR occurrence: muscle toxicity reaction, liver and kidney dysfunction, and digestive tract reaction. The findings from the univariate and multivariate logistic regression analysis conducted on the development group revealed that age ≥ 60 years, body mass index ≥ 23kg/m2, course of disease ≥ 5 years, basic disease type ≥ 3, Dyslipidemia, history of cerebral infarction, high-dose use, and the coadministration of multiple medications were found to be statistically significant independent risk factors for ADR associated with statin use in patients diagnosed with coronary heart disease (P<0.05). A nomogram prediction model was established based on the aforementioned risk factors. |
| Introduction | | | |  |
| Background/rationale | 2 | Explain the scientific background and rationale for the investigation being reported | 3 | CHD has emerged as a prominent contributor to global mortality, particularly prevailing as the leading cause of death among males aged 45 years and above. The clinical manifestations of CHD include chest tightness, difficulty breathing, etc. CHD encompasses a multitude of complications, including angina, arrhythmia, heart failure, thromboembolism, and myocardial infarction. In severe instances, CHD may culminate in cardiac arrest, significantly impinging upon the patient's overall well-being, health status, and quality of life. At present, the medication treatment of CHD is mainly focused on lipid-lowering drugs, among which statins are the preferred drugs for clinical treatment of lipid metabolism disorders. The activity of 3-hydroxy-3-methylglutaryl coenzyme A reductase can be competitively inhibited by statins, thereby reducing the synthesis of endogenous mevalonic acid and cholesterol, and playing a role in regulating lipid. One study have pointed out that, statins are widely used in clinical practice, which can play a role in lowering blood lipids by reducing the level of peripheral lipoproteins in patients with CHD and improving the blockage of stenosis. However, research shows that patients with CHD may experience varying degrees of adverse drug reactions(ADR) after statin therapy, with muscle pain, elevated liver enzymes, and renal function damage being the most common. Therefore, it is of great significance to effectively screen out high-risk groups of CHD patients who experience ADR after taking statins, and to take targeted intervention measures for this group of people for the prevention and treatment of CHD patients. |
| Objectives | 3 | State specific objectives, including any prespecified hypotheses | 3 | This study conducted an analysis on the risk factors associated with ADR in patients diagnosed with CHD who were prescribed statins. Furthermore, a personalized risk prediction model for ADR in CHD patients was developed, aiming to serve as a reference for mitigating the incidence of ADR in CHD patients treated with statins. |
| Methods | | | |  |
| Study design | 4 | Present key elements of study design early in the paper | 4 | The study subjects consisted of 351 patients with CHD who were admitted to the Department of Cardiology of a tertiary hospital in Anhui Province, China from February 2021 to January 2022 and received statin treatment, as identified in a retrospective cohort study. |
| Setting | 5 | Describe the setting, locations, and relevant dates, including periods of recruitment, exposure, follow-up, and data collection | 4 | The retrospective collection of clinical data from research subjects encompassed various demographic and health-related variables, such as gender, age, education level, body mass index (BMI), type of residence, medical expense payment method, hypertension history, presence of Dyslipidemia, smoking habits, alcohol consumption, course of disease, basic disease type, diabetes history, cerebral infarction history, combined medication usage, and high-dose drug utilization. In total, 16 indicators were gathered. Subsequently, the patients were categorized into two groups, namely the ADR group (consisting of 85 cases) and the non-ADR group (comprising 266 cases), based on their experience of adverse drug reactions following the administration of statins. |
| Participants | 6 | (*a*) *Cohort study*—Give the eligibility criteria, and the sources and methods of selection of participants. Describe methods of follow-up  *Case-control study*—Give the eligibility criteria, and the sources and methods of case ascertainment and control selection. Give the rationale for the choice of cases and controls  *Cross-sectional study*—Give the eligibility criteria, and the sources and methods of selection of participants | 4 | Inclusion criteria for research subjects: ①Patients who satisfy the pertinent diagnostic criteria for coronary heart disease (CHD) and have received a diagnosis of CHD via coronary angiography; ②No allergic reactions to statins; ③The patient and their family members have informed consent to the medication treatment plan and precautions; ④Clinical data of subjects is completed without any missing information; ⑤ Patients with good treatment compliance. Exclusion criteria for research subjects: ①Individuals with significant cognitive impairment; ②Individuals diagnosed with malignant neoplasms; ③Patients with organic lesions in important organs such as liver, kidney, and lungs; ④Individuals with hematological or autoimmune diseases; ⑤Patients with missing clinical data. |
|  |  | (*b*) *Cohort study*—For matched studies, give matching criteria and number of exposed and unexposed  *Case-control study*—For matched studies, give matching criteria and the number of controls per case | 4 | Subsequently, the patients were categorized into two groups, namely the ADR group (consisting of 85 cases) and the non-ADR group (comprising 266 cases), based on their experience of adverse drug reactions following the administration of statins. |
| Variables | 7 | Clearly define all outcomes, exposures, predictors, potential confounders, and effect modifiers. Give diagnostic criteria, if applicable | 4 | The retrospective collection of clinical data from research subjects encompassed various demographic and health-related variables, such as gender, age, education level, body mass index (BMI), type of residence, medical expense payment method, hypertension history, presence of Dyslipidemia, smoking habits, alcohol consumption, course of disease, basic disease type, diabetes history, cerebral infarction history, combined medication usage, and high-dose drug utilization. In total, 16 indicators were gathered. Subsequently, the patients were categorized into two groups, namely the ADR group (consisting of 85 cases) and the non-ADR group (comprising 266 cases), based on their experience of adverse drug reactions following the administration of statins. |
| Data sources/ measurement | 8* | For each variable of interest, give sources of data and details of methods of assessment (measurement). Describe comparability of assessment methods if there is more than one group | *5* | *There were 139 (39.60%) female patients and 212 (60.40%) male patients among the 351 study subjects; 238 (67.81%) patients aged ≥ 60 years old among the 351 study subjects; other clinical data on the subjects are described in Table 1. There was no statistically significant disparity observed in the clinical data between the development group and the validation group, as indicated by a P-value greater than 0.05, indicating homogeneity between the two groups.* |
| Bias | 9 | Describe any efforts to address potential sources of bias | 4 | Patients with missing clinical data were excluded. |
| Study size | 10 | Explain how the study size was arrived at | 4 | The study subjects consisted of 351 patients with CHD who were admitted to the Department of Cardiology of a tertiary hospital in Anhui Province, China from February 2021 to January 2022 and received statin treatment, as identified in a retrospective cohort study. |

Continued on next page

| Quantitative variables | 11 | Explain how quantitative variables were handled in the analyses. If applicable, describe which groupings were chosen and why | 5 | To compare the counting data between the two groups, the unadjusted Pearson Chi-square test was employed. |
| --- | --- | --- | --- | --- |
| Statistical methods | 12 | (*a*) Describe all statistical methods, including those used to control for confounding | 5 | Logistic regression was utilized to analyze the risk factors associated with ADR. |
|  |  | (*b*) Describe any methods used to examine subgroups and interactions | Not applicable | Not applicable |
|  |  | (*c*) Explain how missing data were addressed | 4 | Patients with missing clinical data were excluded. |
|  |  | (*d*) *Cohort study*—If applicable, explain how loss to follow-up was addressed  *Case-control study*—If applicable, explain how matching of cases and controls was addressed  *Cross-sectional study*—If applicable, describe analytical methods taking account of sampling strategy | 4 | The patients were categorized into two groups, namely the ADR group (consisting of 85 cases) and the non-ADR group (comprising 266 cases), based on their experience of adverse drug reactions following the administration of statins during hospital. |
|  |  | (*e*) Describe any sensitivity analyses | Not applicable | Not applicable |
| Results | | | | |
| Participants | 13* | (a) Report numbers of individuals at each stage of study—eg numbers potentially eligible, examined for eligibility, confirmed eligible, included in the study, completing follow-up, and analysed | Not applicable | Not applicable |
|  |  | (b) Give reasons for non-participation at each stage | Not applicable | Not applicable |
|  |  | (c) Consider use of a flow diagram | Not applicable | Not applicable |
| Descriptive data | 14* | (a) Give characteristics of study participants (eg demographic, clinical, social) and information on exposures and potential confounders | 5 | Among the 351 study subjects, 85 patients (24.22%) experienced ADR after taking statins, with 152 ADR events. According to the location of ADR, The events were classified into three categories: muscle toxicity, liver and kidney dysfunction, and gastrointestinal reactions. There are three kinds of statins that were taken among the 85 patients: Simvastatin in 20 cases, Atorvastatin in 28 cases, and Rosuvastatin in 37 cases. |
|  |  | (b) Indicate number of participants with missing data for each variable of interest | Not applicable | Not applicable |
|  |  | (c) *Cohort study*—Summarise follow-up time (eg, average and total amount) | Not applicable | Not applicable |
| Outcome data | 15* | *Cohort study*—Report numbers of outcome events or summary measures over time | *5* | *Among the 351 study subjects, 85 patients (24.22%) experienced ADR after taking statins, with 152 ADR events.* |
|  |  | *Case-control study—*Report numbers in each exposure category, or summary measures of exposure |  |  |
|  |  | *Cross-sectional study—*Report numbers of outcome events or summary measures |  |  |
| Main results | 16 | (*a*) Give unadjusted estimates and, if applicable, confounder-adjusted estimates and their precision (eg, 95% confidence interval). Make clear which confounders were adjusted for and why they were included | 6 | The findings from the univariate and multivariate logistic regression analysis conducted on the development group revealed that: age ≥ 60 years, body mass index ≥ 23kg/m2, course of disease ≥ 5 years, basic disease type ≥ 3, Dyslipidemia, history of cerebral infarction, high-dose use, combined medication were independent risk factors for ADR after taking statins in patients with CHD (P<0.05), as shown in Table 2. |
|  |  | (*b*) Report category boundaries when continuous variables were categorized | Not applicable | Not applicable |
|  |  | (*c*) If relevant, consider translating estimates of relative risk into absolute risk for a meaningful time period | Not applicable | Not applicable |

Continued on next page

| Other analyses | 17 | Report other analyses done—eg analyses of subgroups and interactions, and sensitivity analyses | Not applicable | Not applicable |
| --- | --- | --- | --- | --- |
| Discussion | | | | |
| Key results | 18 | Summarise key results with reference to study objectives | 8 | In this study, there were 85 patients who experienced ADR due to the use of statins, with 152 ADR events occurred.  The results of this study showed that the independent risk factors for ADR after taking statins in patients with CHD were age ≥ 60 years, basic disease type ≥ 3, history of cerebral infarction, high-dose use, combined medication, body mass index ≥ 23kg/m^2^, course of disease ≥ 5 years, and dyslipidemia (P<0.05). |
| Limitations | 19 | Discuss limitations of the study, taking into account sources of potential bias or imprecision. Discuss both direction and magnitude of any potential bias | 10 | This study possesses certain limitations. Firstly, it retrospectively examined the risk factors associated with the incidence of adverse drug reactions (ADRs) in patients with coronary heart disease (CHD) following statin usage, and subsequently developed a predictive model based on these findings. However, the study's sample size was small, the number of included indicators was limited, and certain patients were excluded due to incomplete data, potentially introducing bias into the research outcomes. Secondly, both the development group and validation group in this study were derived from the same dataset, thereby relying on relatively singular data sources. If sample data from different time periods or research centers can be added as external validation sets for out-of-phase or multi-center validation, the effectiveness of the model can be further improved.33 Therefore, it is particularly necessary to conduct prospective validation studies with multiple centers, large samples, and more predictive factors in the future. |
| Interpretation | 20 | Give a cautious overall interpretation of results considering objectives, limitations, multiplicity of analyses, results from similar studies, and other relevant evidence | 10 | The incidence of ADR in patients with CHD after taking statins in this study is 24.22%. Regarding ADR events such as muscle toxicity, liver and kidney dysfunction, and gastrointestinal reactions, medical staff should pay attention to prevention and pay more attention. At the same time, based on the eight independent risk factors of age ≥ 60 years old, body mass index ≥ 23kg/m2, course of disease ≥ 5 years, basic disease type ≥ 3, dyslipidemia, history of cerebral infarction, high-dose use, and combined medication, this study constructed a personalized risk prediction model for the risk of ADR, which can screen the CHD patients with high risk of ADR at an early stage, and effectively prevent and intervene against this part of the patients, So as to reduce the incidence of ADR in patients with CHD after taking statins and improve the prognosis of these patients. |
| Generalisability | 21 | Discuss the generalisability (external validity) of the study results | 7 | The discrimination of the nomogram model was assessed using the AUC. In the development group, the AUC of the nomogram model was 0.808 (95% CI: 0.751-0.865), while in the validation group, it was 0.852 (95% CI: 0.752-0.951). These results indicate that the nomogram model exhibits favorable discrimination, as depicted in Figure 3. To evaluate the calibration of the nomogram model, the Hosmer-Lemeshow deviation test was employed. The Hosmer-Lemeshow deviation test results for the nomogram model in both the development group and validation group did not yield statistically significant findings (P>0.05). This suggests that the disparity between the predicted probability of the nomogram model and the observed frequency of ADR in patients was not statistically significant, thereby indicating the satisfactory calibration of the nomogram. This is illustrated in Figure 3. |
| Other information | |  | | |
| Funding | 22 | Give the source of funding and the role of the funders for the present study and, if applicable, for the original study on which the present article is based | 11 | This study was funded by the University-level undergraduate quality engineering project of China University of Science and Technology in 2022 (No.:2022xjyxm089). |

*Give information separately for cases and controls in case-control studies and, if applicable, for exposed and unexposed groups in cohort and cross-sectional studies.

**Note:** An Explanation and Elaboration article discusses each checklist item and gives methodological background and published examples of transparent reporting. The STROBE checklist is best used in conjunction with this article (freely available on the Web sites of PLoS Medicine at http://www.plosmedicine.org/, Annals of Internal Medicine at http://www.annals.org/, and Epidemiology at http://www.epidem.com/). Information on the STROBE Initiative is available at www.strobe-statement.org.
